# Vaccination against hepatitis b virus: are Italian medical students sufficiently protected after the public vaccination programme?

**DOI:** 10.1186/s12995-015-0083-4

**Published:** 2015-11-04

**Authors:** Monica Lamberti, Alfredo De Rosa, Elpidio Maria Garzillo, Anna Rita Corvino, Nicola Sannolo, Stefania De Pascalis, Eliana Di Fiore, Claudia Westermann, Antonio Arnese, Di Giuseppe Gabriella, Albert Nienhaus, Antônio Paulino Ribeiro Sobrinho, Nicola Coppola

**Affiliations:** Department of Experimental Medicine, Section of Hygiene, Occupational Medicine and Forensic Medicine, Second University of Naples, Via dei Crecchi 16, Naples, 80133 Italy; Department of Orthodontics, Second University of Naples, Naples, Italy; Department of Mental Health and Public Medicine, Section of Infectious Diseases, Second University of Naples, Naples, Italy; Institute for Health Services, Research in Dermatology and Nursing, University Medical Centre Hamburg-Eppendorf, Hamburg, Germany; Institution for Statutory Accident Insurance and Prevention in Healthcare and Welfare Services, Hamburg, Germany; Department of Operative Dentistry, Dental School, UFMG - Universidade Federal de Minas Gerais, Belo Horizonte, MG Brazil

**Keywords:** HBV infection, HBV vaccination, Anti-HBs titre, Healthcare students

## Abstract

**Background:**

The development of a vaccine against hepatitis B virus (HBV) has been a major achievement in terms of prevention of HBV infection. For the present study, we analysed the long-term immunogenicity and effectiveness of HBV vaccination among healthcare students with different working seniorities.

**Methods:**

A cross-sectional study of undergraduate and postgraduate students attending the Medical School of the Second University of Naples was conducted between September 2012 and December 2014. HBV serum markers were determined and multivariate logistic regression analysis was used to identify factors associated with the level of long-term immunogenicity.

**Results:**

Of the 2,932 subjects evaluated, only 33 (1.1 %) declared no history of vaccination. All vaccinated subjects were HBsAg/anti-HBc negative, 459 of which had an anti-HBs titre <10 IU/L. The latter were younger, more likely to be attending a healthcare profession school (i.e., dental hygienists, nursing, paediatric nursing, radiography and midwifery) than a medical school (at either undergraduate or postgraduate level) and more likely to have been vaccinated in infancy.

**Conclusion:**

The results of this study suggest that assessment of HBV serum markers in workers potentially exposed to hospital infections is useful to identify small numbers of unvaccinated subjects or vaccinated subjects with low antibody titre, all of whom should be referred to a booster series of vaccinations.

## Background

Hepatitis B virus (HBV) can cause dreadful infectious diseases and, as such, is a major global health problem [1]. Infection can be asymptomatic or symptomatic, can take the form of acute or chronic liver disease and is potentially fatal. The World Health Organisation (WHO) estimates that, globally, about 2 billion people have been infected with HBV, more than 350 million are chronically infected, and nearly one million per year die from its acute or chronic sequelae [2]. The risk run by healthcare personnel (HCP) of contracting HBV is four times greater than that of the general adult population [3]. The most common routes of transmission from patients to HCP are needlestick and other sharps injuries, followed by mucocutaneous exposure [4]. The risk of transmission of the virus per needlestick injury from a patient infected with HBV is 37–62 %, among the highest risk rates for a virus [5].

In addition to HCP, students and assistants in training are also potentially exposed to hospital infections [6]. A range of different measures and interventions, such as the use of safety devices, has helped to reduce the risk of HBV transmission to HCP, but the development of HBV vaccines must be considered the major achievement in terms of prevention of HBV infection [4].

Since 1991, there has been a compulsory vaccination programme in Italy for infants: the procedure is reported on a vaccination card, a document designed exclusively to record basic identifying information and the immunization services received. The vaccination coverage has been high (94 %), and the programme has greatly reduced the incidence of HBV infection [7]. All babies are vaccinated (at month 3, 5 and 11) to prevent the risk of acquiring HBV infection by perinatal and familial transmission; moreover, during the first 12 years of application of the programme, all children aged 12 were also vaccinated to prevent HBV transmission by unsafe sexual activity or intravenous drug use. This strategy permitted the programme to cover the Italian population aged 0–24 years by 2003 [8].

It is generally observed that antibody titres decline over time following immunization, resulting in an increased rate of infection, especially when the titre of the antibody to hepatitis B surface antigen (HBsAg) falls below the protective cut-off level of ≥10 mIU/mL [9]. The present study was therefore carried out to evaluate the long-term immunogenicity and effectiveness of the Italian HBV vaccination programme in students attending the medical and healthcare schools of the Second University of Naples, Italy, and to identify independent predictive factors of long-term immunogenicity.

## Methods

This study was conducted at the Second University of Naples, Naples, Italy, from September 2012 to December 2014. Based on the risk assessment document, during this period we carried out a health surveillance programme of third and sixth year medical school students, first and third year health profession school students (three-year nursing, dental hygienists, paediatric nursing, radiography and midwifery courses), and first and third year students of the university’s specialisation schools (all of whom were post-medical school students). All were actively recruited before any possible exposure to risky procedures or patients connected with the educational path, as established by our health protocol [10, 11]. HBV serum markers (HBsAg, anti-HBs and total anti-HBc) were determined at year 3 for the medical school students, and year 1 for students of the health profession schools and of the specialisation schools. HBsAg titre was re-evaluated again at a later time only in subjects with a low (<10 IU/L) anti-HBs titre.

After obtaining informed written consent, all students visited for the first time were asked to complete a pre-coded questionnaire stating their age, gender, hepatitis B vaccination status, previous exposure to HBV and educational level. The information on HBV vaccination given by the students was always checked against their vaccination cards. A blood sample was taken from each participant under strict aseptic conditions in a plain vaccutainer. Blood was allowed to clot and serum was separated and stored at *−*20* °*C until testing. HBV serum markers (HBsAg, anti-HBs and total anti-HBc) were determined using commercial immunoenzymatic assays (Abbott Laboratories, North Chicago, IL, USA). Anti-HBs titres were extrapolated from a calibration curve generated using the WHO reference standard and are expressed in IU/L. In particular, actual values were obtained for anti-HBs titres between 10 and 400 IU/mL, and for this interval the geometric mean was calculated using standard procedures; for values <10 IU/mL or >400 IU/mL, the laboratory readout indicated only either “under 10 IU/mL” or “over 400 IU/mL”, respectively.

According to Italian legal guidelines for observational studies, ethical approval for conducting this survey was unnecessary and, accordingly, cross-sectional studies did not require formal approval by local institutional review boards [12]. Personal information on the subjects included in the study was protected according to Italian law [13]. Statistical analysis was performed with StatGraph v3.0. Continuous variables are given as mean±standard deviation, and categorical variables as the absolute value and relative frequency. Differences in mean were evaluated using an unpaired Student t-test, and the chi-squared test was applied to categorical variables. A *p*-value of <0.05 was considered to be statistically significant.

## Results

In total, 2,932 students attending the medical school, the healthcare profession schools and the specialisation schools of the Second University of Naples were screened from September 1, 2012, to December 31, 2014. On the questionnaire, 2,899 (98.9 %) students declared a history of HBV vaccination. The remaining 33 (1.1 %) were not born in Italy: 32 were HBsAg/anti-HBs/anti-HBc negative, and one was HBsAg positive; 23 were attending a healthcare profession school, seven the medical school and three were specialising doctors. All were being evaluated for HBV for the first time.

The HBsAg positive subject was attending the first year of the thoracic surgery specialisation school, and on the advice of the infectious disease specialist, was started on a course of antiviral therapy and included in a follow-up programme for the monitoring of viral load. The student returned to work under the obligation of using personal protective equipment (Kevlar cut-resistant gloves), had to present at the customary medical check-ups with a reduced periodicity of six months, and was temporarily prohibited from carrying out any highly invasive surgery.

The 2,899 vaccinated subjects were all HBsAg/anti-HBc negative. One thousand seven hundred and forty one students (60.1 %) were female and 2,868 (98.9 %) were of a Caucasian Italian ethnic background; 994 had received a course of 3 paediatric doses (10 μg) of recombinant hepatitis B vaccine at their 3rd, 5th and 11th month of postnatal life, and 1,905 had received a course of 3 adult doses (20 μg) of the same vaccine when 12 years old. The majority (84.2 %) of the students had an anti-HBs titre >10 IU/L. Only 362 students were sure that their mothers were HBV negative at the time of giving birth to them (Table 1).

**Table 1:** Demographics of vaccinated, HBsAg-negative students

Characteristic		Value
No. of subjects		2,899
Age in years		26 ± 4.8
Female Reported HBV status of mothers		1,741 (60.1) 362
Place of birth:	Italy	2,868 (98.9)
	Elsewhere	31 (1.1)

Continuous variables are given as mean±SD; categorical variables are given as n° (%)

Compared with students vaccinated at age 12 years, those vaccinated in infancy were younger (21.5 ± 1.0 vs. 28.0 ± 4.5, *p* <0.000), more frequently female (72 % vs. 54 %, *p* <0.000) (Table 2) and had a significantly lower prevalence of individuals with a low (<10 IU/L) anti-HBs titre (12.3 vs. 22.6 %) (Table 2).

**Table 2:** Characteristics of the 2,899 vaccinated students stratified by age at vaccination

		Vaccinated in infancy	Vaccinated at age 12 years	*p*
No. of students		994	1,905	
Age in years		21.8 ± 1.0	28.0 ± 4.5	0.000
Females		715 (72)	1,026 (54)	0.000
Place of birth:	Italy	987 (99.3)	1,881 (98.7)	n.s.
	Elsewhere	7 (0.7)	24 (1.3)	
Attending:	School of medicine	56 (5.6)	839 (44)	0.000*
	Healthcare profession school	938 (94.4)	468 (24.6)	
	Postgraduate medical school	0	598 (31.4)	
Years since vaccination		22.1 ± 1.1	16.1 ± 3.1	0.000
Students with HBsAb <10IU/L		224 (22.6)	235 (12.3)	0.000
Students with HBsAb >10 IU/L		770 (77.4)	1670 (87.7)	0.000

*Healthcare professions school students vs. all medical school students (graduate + postgraduate)

Continuous variables are given as mean±SD; categorical variables are given as n° (%)

**Fig. 1:**
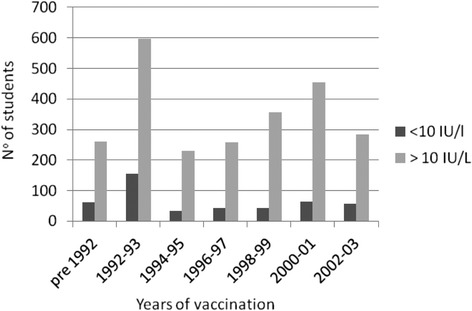
Number of vaccinated students with an anti-HBs titre <10 IU/L or >10 IU/L, stratified by year of vaccination

The subjects with an anti-HBs titre <10 IU/L were younger, more frequently attending a healthcare profession school, and had more frequently been vaccinated in infancy (Table 3 and Fig. 1). The 32 unvaccinated HBsAg/anti-HBs/anti-HBc negative subjects were required to undergo the complete HBV prophylaxis programme.

**Table 3:** Characteristics of students stratified by anti-HBs titre

		<10 IU/L	>10 IU/L	*P*
No. of students		459(15.8)	2,440 (84.2)	
Age in years		24 ± 5.3	26 ± 5.1	0.000
Females		269 (58.6)	1,472 (60.3)	n.s
Attending:	School of medicine	112 (24.4)	792 (32,5)	0.0001*
	Healthcare professions school	254 (55.3)	1,175 (48.1)	
	Postgraduate medical school	93 (20.3)	473 (19.4)	
Vaccinated	in infancy	235 (51.2)	759 (31.1)	0.000
	at age 12 years	224 (48.8)	1,681 (68.9)	

*Healthcare profession school students vs. all medical school students (graduate + postgraduate). Continuous variables are given as mean±SD; categorical variables are given as n° (%)

## Discussion

In 2013, the Integrated Epidemiological System for Acute Viral Hepatitis (SEIEVA) reported an incidence of HBV infections in Italy of only 1 per 100,000 individuals; about 20 % of acute HBV infections concerned immigrants, especially from Eastern Europe and Africa, areas highly endemic for HBV [14]. This large reduction in the incidence of HBV infection is certainly also linked to the vaccination programme for infants and 12-year-old adolescents in Italy since 1991.

No student that had been vaccinated was found to have been infected with HBV, as reflected by the absence of positivity to serum HBsAg or anti-HBc after a mean period of nearly 18 years. Thus, the efficacy of the programme is excellent (i.e., 100 %). However, we found that factors such as older age decreased immunogenicity, and that low levels of anti-HBs could be predictive of non-protection. Other factors, such as smoking and obesity, not assessed in our study, may also be involved in decreased immunogenicity [15–17].

Several studies have reported that 85–90 % of those vaccinated as adolescents have anti-HBs levels >10 mIU/mL when measured 10 years after vaccination. This percentage was 40–60 % for those vaccinated as infants, as measured 15–20 years after vaccination [18, 19]. The possible causes of the low anti-HBs titres in adult subjects vaccinated in infancy could be a lower interaction between B and T cells in babies, and in some cases, the presence of serum anti-HBs in mothers might affect the response to the HBV vaccine in babies [20, 21]. This is coherent with our finding that 87.7 % of students vaccinated during adolescence had an anti-HBs titre >10 IU/L, while only 77.4 % of those vaccinated in infancy had a titre >10 IU/L. Closer analysis of our results revealed that healthcare profession students, who were mainly female, had a higher percentage of individuals with an anti-HBs titre <10 IU/L. Indeed, this group was the youngest (they were assessed during their first year of studies, rather than during the third year, as done for the medical students, as required by the risk assessment document) and therefore had the highest number of subjects who had received compulsory vaccination in infancy.

HCP and healthcare students are reported to have the highest occupational risk for HBV infection, with approximately 66,000 HBV infections per year worldwide. Therefore, testing medical students for anti-HBs levels may be warranted as they represent a high-risk population [22–24]. This is particularly relevant in countries where HBV vaccination programmes for infants and adolescents has been introduced but no post-vaccination serological testing is done, making it impossible to identify unvaccinated subjects [25, 26]. In our group, 33 students had not been vaccinated, one of whom had acquired an HBV infection. Italian law (Article 279 of Legislative Decree n. 8, 19 April 2008) requires only to review the vaccination status of HCP and healthcare students prior to employment or to enrolment in a course with a documented possible risk of HBV contagion. However, the decree currently only recommends that unvaccinated individuals be vaccinated; in other words, there is no clear legal obligation to do so. The vaccination procedure is charged to the employer, and the occupational physician must perform the vaccination procedure [27], but even if the non-vaccination of an unvaccinated worker does not lead to automatic discharge, it may lead the occupational physician to limit activities deemed risky.

In our case, all 33 unvaccinated subjects were sent for vaccination and re-evaluation of anti-HBs titre. The HBsAg positive subject was put on antiviral therapy and was included in a follow-up programme monitoring viral load.

In HCP and healthcare students, it is necessary to assess anti-HBs titre either at matriculation or at the start of employment in order to identify subjects with levels <10 IU/L. In fact, although the current WHO view is that subjects with an anti-HBs titre <10 IU/L still retain memory immunity, and no booster dose is necessary as part of a routine immunization programme [27], a more protective approach should be adopted for healthcare workers under continuous risk of exposure to HBV [28, 29]. To distinguish between non-responsive subjects and those whose titres have waned over time but who are still protected, a ‘challenge’ or ‘booster’ dose should be administered and titres rechecked 1–2 months later. Those presenting with titres ≥10 IU/L can be considered protected and not needing additional testing or vaccination, whereas those presenting with titres that are still <10 IU/L after the challenge dose should be referred to a vaccination programme. Indeed, previous studies of young subjects who had been vaccinated in infancy have indicated that a single-dose booster may not reliably induce an anti-HBs response, suggesting that humoral memory immunity reduces with time. Lu CY et al. showed that the HBsAg-specific T cell immune response was negative in 27.2 % of subjects who had received a booster 15 to 18 years after HBV vaccination in infancy [30]; a more recent study of young adults (ages ranging from 18.6 to 20.5 years) who had been immunized against HBV as infants reported that nearly 20 % of subjects with HBsAb <10 IU/L failed to respond to a booster injection [31].

## Conclusion

The present study evaluates the efficacy and immunogenicity of HBV vaccination in a large series of subjects with a high risk of being exposed to HBV*.* The results of this study suggest that assessment of HBV markers is worthwhile to identify the small number of medical students and HCP that may not have been vaccinated, such as immigrants from countries without universal immunisation, and HBV carriers that exist for various reasons, including rare failure in protection or uptake after immunisation.

## Abbreviations

HBV: hepatitis B virus
WHO: World Health Organisation
HBsAg: HBV surface antigen
HCP: healthcare personnel
