# Erratum to: Vaccination against hepatitis b virus: are Italian medical students sufficiently protected after the public vaccination programme?

**DOI:** 10.1186/s12995-016-0092-y

**Published:** 2016-02-15

**Authors:** Monica Lamberti, Alfredo De Rosa, Elpidio Maria Garzillo, Anna Rita Corvino, Nicola Sannolo, Stefania De Pascalis, Eliana Di Fiore, Claudia Westermann, Antonio Arnese, Gabriella Di Giuseppe, Albert Nienhaus, Antônio Paulino Ribeiro Sobrinho, Nicola Coppola

**Affiliations:** Department of Experimental Medicine, Section of Hygiene, Occupational Medicine and Forensic Medicine, Second University of Naples, Via dei Crecchi 16, Naples, 80133 Italy; Department of Orthodontics, Second University of Naples, Naples, Italy; Department of Mental Health and Public Medicine, Section of Infectious Diseases, Second University of Naples, Naples, Italy; Institute for Health Services, Research in Dermatology and Nursing, University Medical Centre Hamburg-Eppendorf, Hamburg, Germany; Institution for Statutory Accident Insurance and Prevention in Healthcare and Welfare Services, Hamburg, Germany; Department of Operative Dentistry, Dental School, UFMG - Universidade Federal de Minas Gerais, Belo Horizonte, MG Brazil

Unfortunately, the original version of this article [1] contained an error. The name of one of the authors was included incorrectly and it read “Di Giuseppe Gabriella” instead of “Gabriella Di Giuseppe”.

The name has been correctly included in full in this erratum.
